# *Mycobacterium wolinskyi* Infection After Local Facial Injection: A Two‐Case Report With Therapeutic Comparison

**DOI:** 10.1111/jocd.71248

**Published:** 2026-10-14

**Authors:** Yonghao Xu, He Wen, Jiahui Li, Wei Li

**Affiliations:** ^1^ Department of Dermatology Qilu Hospital of Shandong University Jinan China; ^2^ Department of Clinical Laboratory Qilu Hospital of Shandong University Jinan China

To the Editor,

*Mycobacterium wolinskyi* infection is rare. Of the few dozen reported cases, most occurred secondary to surgical procedures or similar factors. The treatment typically involves surgical intervention and a combination of two or more sensitive antibiotics administered over several months. Rifampicin is generally not recommended for this infection due to common drug resistance. Herein, we describe two patients, one of whom was successfully cured with rifampicin combined with minocycline.

A 42‐year‐old woman and her 11‐year‐old son both presented with multiple facial abscesses 1 month after receiving nonstandard subcutaneous injections at an unlicensed medical clinic (Figure 1). The child's lesions had received local incision and drainage combined with piperacillin‐tazobactam treatment, yet the regimen proved ineffective. Diagnosis was confirmed through histopathological examination, metagenomic next‐generation sequencing, and whole‐genome sequencing, identifying *M. wolinskyi* . The combination of minocycline and co‐trimoxazole was administered but yielded no therapeutic response in either patient. Drug susceptibility testing identified the isolate as susceptible to moxifloxacin, minocycline, linezolid, amikacin, and ethambutol, and resistant to clarithromycin, tobramycin, and sulfamethoxazole. The rifampicin MIC was 4 mg/L. The treatment regimen was adjusted to minocycline combined with moxifloxacin for the mother and minocycline plus rifampicin for the boy. Both patients achieved complete resolution of skin lesions after 22 weeks of therapy, with no recurrence during 1 year of follow‐up; the mother additionally underwent surgical excision of residual scar tissue (Figure 2).

**FIGURE 1:**
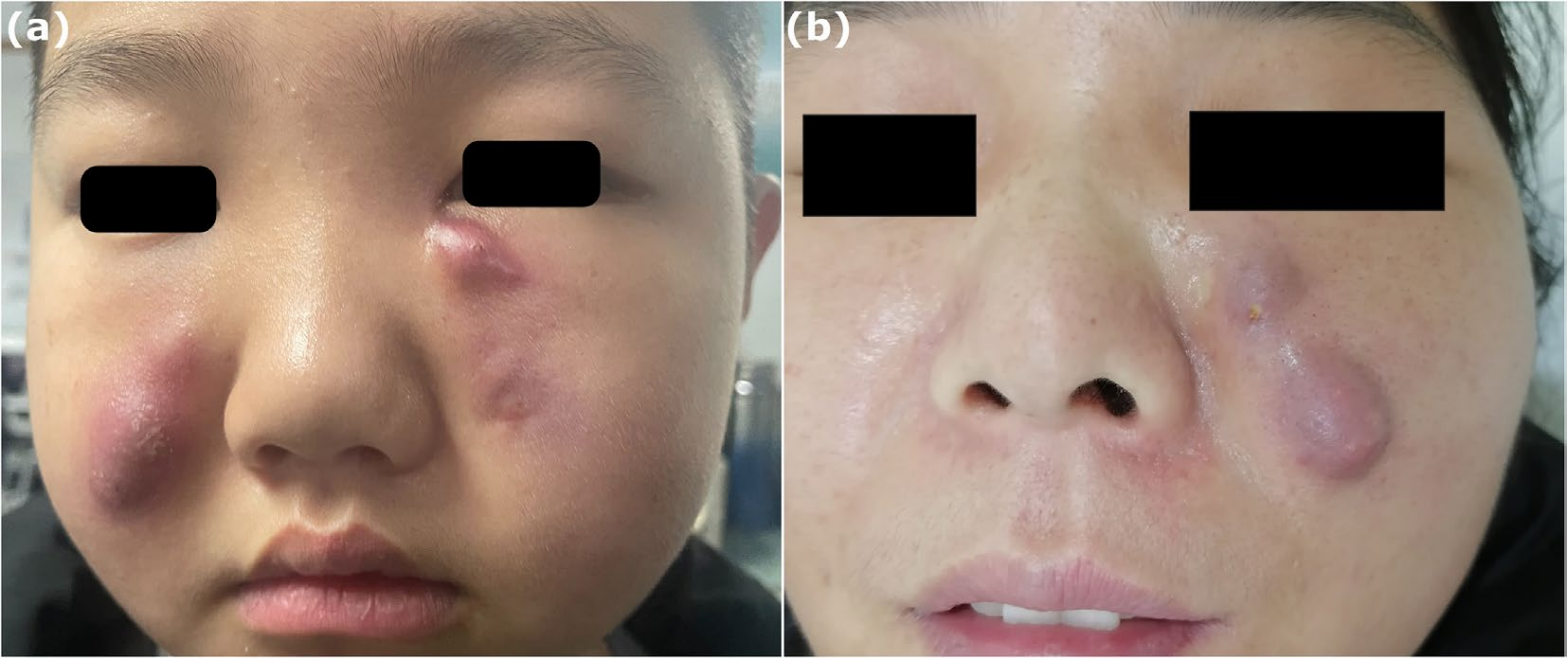
Skin lesions in the boy (a) and his mother (b) at initial presentation.

**FIGURE 2:**
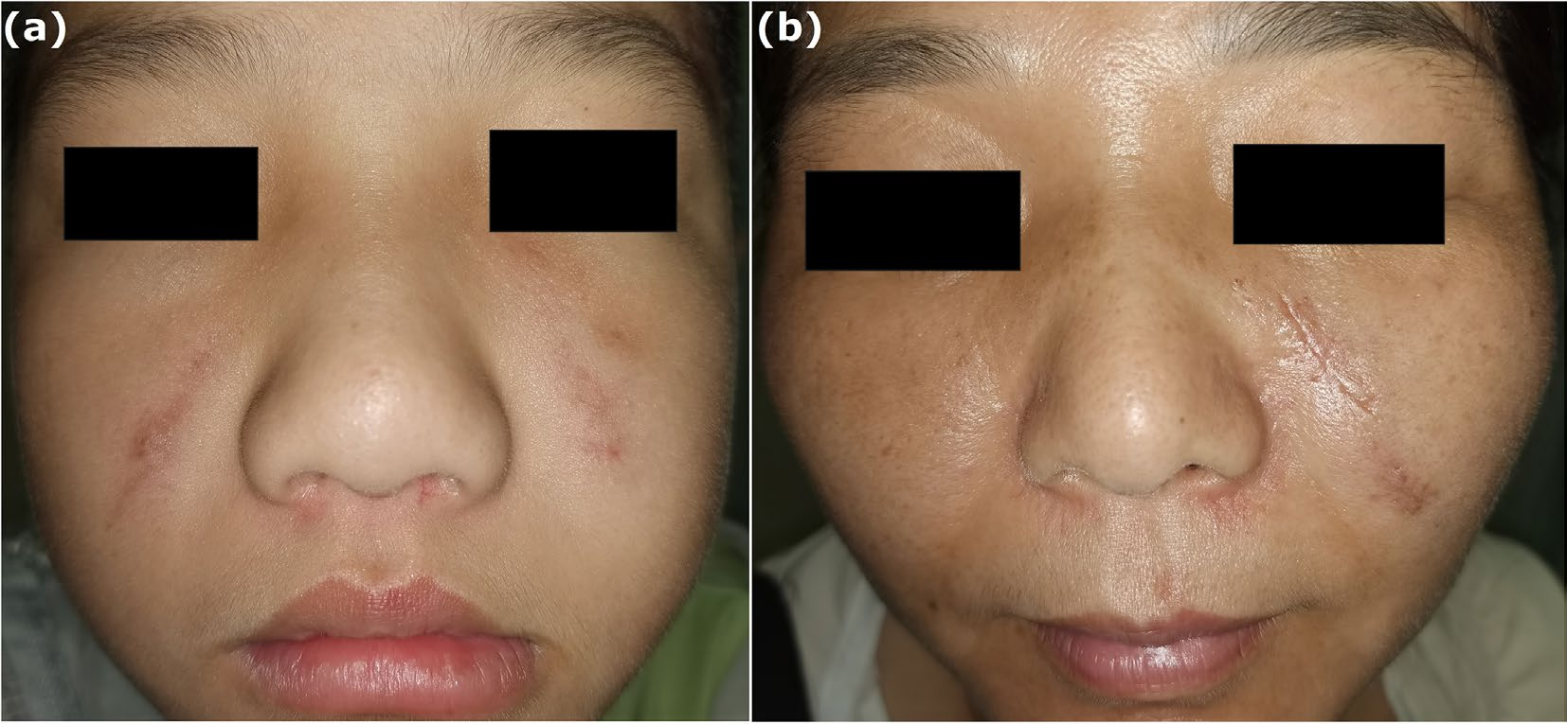
Complete resolution of skin lesions in the boy (a) and his mother (b) after 22 weeks of treatment.

*M. wolinskyi* can infect the heart and aorta, bones and joints, peritoneum, skin and soft tissues, cornea, and respiratory tract, and hematogenous dissemination may even occur. The majority of documented cases are attributed to surgical or traumatic predisposing events. The two patients in our study developed infections after receiving injections from the same medication batch, yet no comparable infectious episodes were identified among other recipients of this batch. It suggests that their infections were likely attributable to substandard aseptic practices during injection administration.

Surgical debridement or prosthetic removal is often critical for resolution of *M. wolinskyi* infection. In our pediatric case, early surgical intervention was performed, yet the infection recurred in the absence of effective concurrent antimicrobial therapy. Among the five susceptible antimicrobial agents, moxifloxacin and minocycline formed a safe, convenient, and highly cost‐effective combination and were therefore selected as the first‐line regimen for the mother. Given the safety limitations of moxifloxacin, amikacin, and ethambutol in pediatric populations, we opted for rifampicin combined with minocycline for the child after obtaining parental consent. This regimen yielded therapeutic efficacy comparable to the moxifloxacin–minocycline combination, despite a rifampicin MIC of 4 mg/L. By contrast, the initial regimen comprising minocycline plus co‐trimoxazole was ineffective in this patient.

The boy achieved full recovery, which may be attributable not only to surgical drainage, but also to several pharmacological factors. *M. wolinskyi* is a rapidly growing nontuberculous mycobacterium (RGM), characterized by poor intrinsic susceptibility to rifampicin. Notably, heterogeneity in rifampicin susceptibility exists among RGM species; for instance, the *Mycobacterium chelonae* type strain ATCC 35752 displays in vitro rifampicin susceptibility [1]. To our knowledge, this is the first report documenting successful treatment of *M. wolinskyi* infection with a rifampicin–minocycline combination. This case underscores that clinical decision‐making for rifampicin‐based management of *M. wolinskyi* infections should not rely exclusively on in vitro susceptibility results, as the correlation between such in vitro results and clinical efficacy remains unclear. Rifampicin exhibits unique pharmacokinetic properties, including high accumulation within inflammatory and necrotic tissues, enabling it to achieve bactericidal concentrations at sites of infection—a feature not shared by moxifloxacin [2]. Moreover, the rifampicin–minocycline combination might exert synergistic activity here, as described for *Mycobacterium tuberculosis* [3]. This low‐cost and well‐tolerated regimen may be considered a potential option for carefully selected individual cases. Nevertheless, given that it was only administered to a single patient and clinical data regarding pediatric *M. wolinskyi* infection remain extremely limited, large‐scale clinical studies and further clinical evidence are required to validate the overall efficacy and synergistic mechanism of this combined therapy, and it is inappropriate to extend this conclusion to all patients with *M. wolinskyi* infection.

## Funding

The authors have nothing to report.

## Ethics Statement

This study was conducted in accordance with the ethical principles outlined in the Declaration of Helsinki.

## Consent

Written informed consent was obtained from all enrolled patients. Ethical approval was granted by the Scientific Research Ethics Committee of Qilu Hospital, Shandong University.

## Conflicts of Interest

The authors declare no conflicts of interest.

## Data Availability

The raw clinical data are not publicly available due to patient‑privacy and ethical restrictions. De‑identified data may be available upon reasonable request from the corresponding author with appropriate ethical approval.
